# Comment on ‘Neutrophils: driving progression and poor prognosis in hepatocellular carcinoma?’

**DOI:** 10.1038/s41416-018-0241-4

**Published:** 2018-09-12

**Authors:** Yuying Liu, Xianchun Meng

**Affiliations:** 1 Physical Examination Department, First Affiliated Hospital of Zhengzhou University, Zhengzhou, Henan 450052 China; 2 Clinical Laboratory, First Affiliated Hospital of Zhengzhou University,Key Laboratory of Laboratory Medicine of Henan province, Zhengzhou, Henan 450052 China

**Keywords:** Biomarkers, Medical research

We read with great interest the article by Margetts et al.^1^ The authors provided clinical evidence that there is a potential correlation between neutrophils and disease progression and poor prognosis of hepatocellular carcinoma. In both UK and Hong Kong cohorts, neutrophils, platelets, lymphocytes, the neutrophil/lymphocyte ratio (NLR) and the systemic immune-inflammation index (SII) correlated stepwise, either increasing or decreasing (lymphocytes), with tumour node metastasis (TNM) and Childs-Pugh stage, performance status (PST) and consequently with the combined Barcelona Clinic for Liver Cancer (BCLC) stage. Survival analyses confirmed the NLR and SII as highly significant prognostic biomarkers. Furthermore, the authors observed that, when focused on individual cell types, only the neutrophil count was independently associated with both TNM stage and PST, as well as being significantly and independently associated with poorer survival, which means in this study of 1168 patients, neutrophils alone, rather than lymphocytes or platelets, were independently associated with outcome. However, some points of the manuscript warrant discussion.

First, despite there being no significant survival difference between the two patient cohorts, differences of demographics (race, age and gender), tumour characteristics (tumour size, extrahepatic disease, vascular invasion and cirrhosis), aetiology of liver disease, ECOG PS, TNM, Childs-Pugh, BCLC stage, and primary treatment between the two groups were all statistically significant, which were all potentially or authentically associated with a change of peripheral blood cells.^2–5^ Studies have confirmed that race, age and gender cause physiological differences of peripheral blood cells.^2^ Furthermore, in our recent study, we defined reference intervals for NLR and SII. We found that for NLR there was a requirement for age and gender stratification; however, this was not the case for SII.^4^ Although, the present study provided the opportunity to explore the prognostic value of peripheral cell counts and combination scores in much greater depth than before, direct mixing of data from two obviously heterogeneous cohorts is not consistent with statistical rules and may lead to wrong conclusions.

Second, peripheral blood cell counts of healthy people are influenced by many factors, such as race, gender, age, and infection, amongst others. Furthermore, our previous study found that in patients with different diseases, or even with different stages of the same disease, changes of the peripheral blood cell count may be different. Three numerical types of pre-procedural observed values, namely higher, lower, or no great change, compared with healthy controls, can be found; these could all be a source of variation.^6^ In patients with a disease such as liver cancer, peripheral blood cell count can be changed by either physiological or pathological reasons. Although distinguishing the changes of peripheral blood cell count caused by various factors is extremely complex, only achieving clear relationships between these changes and disease progression and treatment effect can guarantee the authenticity and accuracy of the final conclusion.

Third, traditional clinical research usually involves conducting comparative analysis among groups with different interventions. Even though demographic factors may confirm no statistical differences, many other factors that may affect the result of the intervention are often ignored. The development of information technology makes the storage, acquisition and management of data much more convenient and efficient. Statistical analysis of data based on large sample sizes should be more reasonable in selecting relevant statistical methods. The traditional data analysis methods based on small sample size are often not suitable for large sample size data analysis.^7^ However, this paper analyzed data from two cohorts with large sample volumes and with lots of unmatched conditions, while differences of factors such as the race, age, gender are all potential or direct interferences of peripheral blood cells. Although, the data for each group are accurate and reliable, directly comparing data from two groups, and then combining the data from two groups directly for further comparative analysis without eliminating unmatched conditions ignores the physiological differences and data heterogeneity of the two large cohorts. The analysis methods may have large issues that would make the reliability of the experimental conclusion challenging.

Finally, for data analysis of a large sample size, propensity score matching (PSM) is an excellent statistical method to eliminate the mismatch between groups.^8^ For the experimental design scheme of this paper, conditions were matched with a 1:1 ratio to ensure consistency and to ensure the homogeneity of several potential interference factors. Thus, conclusions based on data of the matched groups will be more reliable and persuasive. Of course, screening and validation of the characteristic indexes in the large sample data by statistical methods such as Decision Tree and Random Forest will be quicker and more effective.^7^

In conclusion, the dilemma still persists of whether neutrophils alone, rather than lymphocytes or platelets, are independently associated with disease progression and poor prognosis of hepatocellular carcinoma, or whether this is a surface phenomenon caused by inappropriate data processing methods. Future studies are needed to assess this, and we believe statistical methods that are not limited to data distribution and more suitable for analysis of large sample sizes, such as Propensity Score Matching and Decision Tree and Random Forest, will greatly improve the reliability of medical studies in the big-data era, and may promote the realization of the precision medicine.
